# DNA Barcoding of the parasitoid wasp subfamily Doryctinae (Hymenoptera: Braconidae) from Chamela, Mexico

**DOI:** 10.3897/BDJ.3.e5109

**Published:** 2015-05-18

**Authors:** Daniela Gutiérrez-Arellano, Claudia Renata Gutiérrez-Arellano, Alejandro Zaldívar-Riverón

**Affiliations:** ‡ Colección Nacional de Insectos, Instituto de Biología, Universidad Nacional Autónoma de México, 3er. circuito exterior s/n, Cd. Universitaria, Copilco Coyoacán, A. P. 70-233, C. P. 04510, D. F., Mexico; § Museo de Zoología “Alfonso L. Herrera”, Facultad de Ciencias, Universidad Nacional Autónoma de México, Apartado Postal 70-399, C. P. 04510, D. F., Mexico

**Keywords:** Ichneumonoidea, species richness, Neotropics, faunistic study, COI

## Abstract

*Background and aims*. The Doryctinae is a considerably diverse, poorly studied group of parasitoid wasps and one of the most diverse subfamilies within Braconidae. Taxonomic knowledge of this group remains highly incomplete, specially in the tropics. In Mexico, it has been reported as the subfamily with the highest number of recorded genera. A preliminary Barcoding study carried out in the Chamela region, located near the Mexican pacific coast in Jalisco, identified 185 barcoding species of Dorytinae assigned to 19 identified doryctine genera. This work updates the later study, representing a three years effort to assess the species richness of this subfamily for the Chamela region.

*Materials and methods*. Ten collecting field trips of 5 to 10 days each were carried out from June 2009 to May 2011. A 2% divergence criterion using the BIN system implemented in BOLD was followed in order to establish species boundaries among the specimens that were collected.

*Results and conclusions*. A total of 961 specimens were collected, from which 883 COI sequences were obtained. The sequences generated corresponded to 289 barcoding species and 30 identified genera. The most speciose genera were *Heterospilus* Haliday (170 spp.), *Ecphylus* Förster (19 spp.), *Allorhogas* Gahan (15 spp.) and *Callihormius* Ashmead (14 spp.). Addition of previously collected material increased the diversity of the subfamily in the region to 34 genera and 290 species. Paraphyly of *Heterospilus* with respect to *Neoheterospilus* and H*eterospathius* was again recovered. Twenty new species and two new genera (*Sabinita* Belokobylskij, Zaldívar-Riverón et Martínez, *Ficobolus* Martínez, Belokobylskij et Zaldívar-Riverón) have been described so far from the material collected in this work.

## Introduction

Biodiversity inventories represent an integral component for the adequate management of natural resources of any country. In the case of faunistic studies, however, these rarely include arthropod taxa due to their considerably high diversity and scarce taxonomic knowledge. In this context, DNA Barcoding (Hebert et al. 2003a) represents a fast, valuable approach to built species inventories of highly diverse, poorly known invertebrate groups.

With over 1,300 described and almost 3,000 estimated species, Doryctinae is one the four most diverse subfamilies of braconid parasitoid wasps together with the Braconinae, Microgastrinae and Opiinae (Jones et al. 2009, Yu et al. 2012). Doryctine wasps are mainly known to be idiobiont ectoparasitoids of xylophagous and bark boring coleopteran larvae, though other host groups (e.g. Lepidoptera, Diptera, Hymenoptera) and biologies (e.g. phytophagy, endoparasitoidism) have also been reported (Belokobylskij 1992, Belokobylskij et al. 2004). Several doryctine species are known to be parasitoids of insect pests (LaSalle and Gauld 1993), therefore they play an important role for maintaining the balance of terrestrial ecosystems (Hawkins and Hochberg 1994).

The taxonomic knowledge of the Doryctinae is still far from complete, especially in the tropics and subtropics, where most of its species richness is known to occur (Belokobylskij 1992). In the Mexican territory, this was recently reported as the subfamily with the highest number of recorded genera (63 genera; Coronado-Blanco and Zaldívar-Riverón 2014). However, this number contrasts with its scarce number of described species that have been reported for the country. Recently, Zaldívar-Riverón et al. (2010) showed the preliminary results of a DNA Barcoding survey of the Doryctinae from Chamela Biological Station (CBS) in Mexico, which located near the Pacific coast in the state of Jalisco and is mainly composed of tropical dry forest (Noguera et al. 2002). Zaldívar-Riverón et al. (2010)​ not only revealed and extraordinary, mostly undescribed species richness for this group (185 Barcoding and 20 recognized genera), but also showed some previously undetected taxonimic problems.

Here we show the final list of the DNA Barcoding species inventory of the Doryctinae from the CBS, which was carried out during three years of collecting effort. We updated the list of genera that occur in the region and show their number of DNA Barcoding species based on the barcoding index criterion (BIN; Ratnasingham and Hebert 2013). This faunistic study highlights the extraordinary, neglected species richness of this parasitoid group in the Mexican territory, and is also serving as a basis for the subsequent description of several new taxa.

## Materials and methods

### Study area

**Figure 1.**
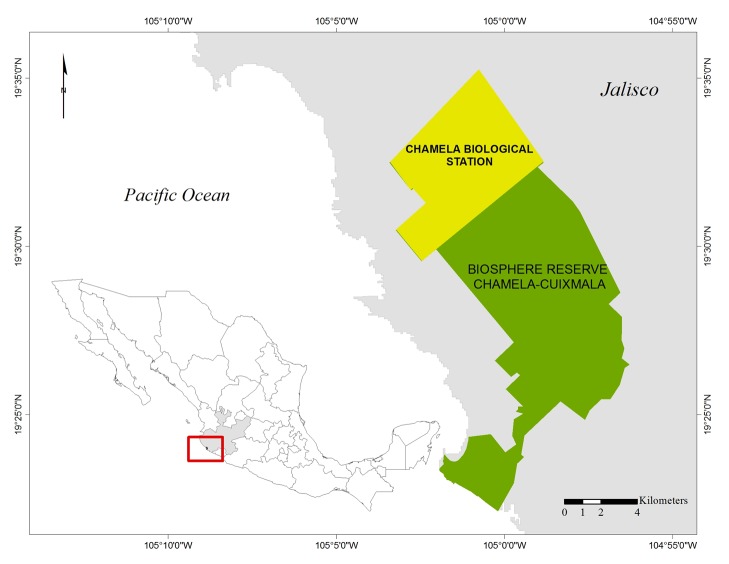
Study area. The Chamela Biological Station (IB-UNAM), located within the Chamela-Cuixmala Biosphere Reserve in the estate of Jalisco, Mexico.

Specimens belonging to the subfamily Doryctinae were collected in the Chamela biological station (Fig. 1), owned by the Instituto de Biología, Universidad Nacional Autónoma de México (UNAM). This station is located within the Chamela–Cuixmala Biosphere Reserve (CCBR), near the Pacific coast in the state of Jalisco(19°29' N, 105° 02' W). The prevailing climate in the area is tropical sub-humid (mean annual temperature 24.6 °C, annual precipitation 788 mm; García 1988). There is a strong seasonality in the region, with alternating rainy (Jul- Oct) and dry (Nov-Jun) seasons. Altitude ranges from 0 to 500 meters above sea level. The dominant type of vegetation is tropical dry forest, which is characteristic of the Mexican Pacífic coast, though patches of tropical evergreen forest, coastal dune, xeric shrubland and mangrove are also present (Rzedowski 1978, Noguera et al. 2002). The relevance of this area lies in the variety of habitats it supports, as well as in its high endemic component of plants, vertebrates and insects (Noguera et al. 2002).

### Specimen sampling

A total of 10 collecting trips of 5 to 10 days each were carried out from June 2009 to May 2011. Collects were performed both during the rainy and dry seasons in order to have a complete representation of the examined group through the year. Specimens were collected in 24 sites within the CBS boundaries (Table 1), using for this: 1) malaise traps, 2) light traps, 3) yellow pan traps, as well as 5) sweep nets for at least 4 h per day (Marshall 1994). The collected materials were preserved in 96% ethanol and kept at -20 °C. All specimens were sorted out at subfamily level following the taxonomic key of New World genera of Braconidae (Wharton et al. 1997). Doryctine specimens were then identified to genus level using the relevant literature (Marsh 1997, Marsh 2002) and subsequent descriptions of genera (e.g. Zaldívar-Riverón et al. 2014).

### DNA sequencing

DNA samples were obtained from a single hind leg and sent for DNA extraction and amplification to the Canadian Center for DNA Barcoding at University of Guelph, Ontario (see detailed laboratory protocols in Smith et al. 2008). A 615–658 bp fragment corresponding to the standard animal DNA barcoding locus (cytochrome c oxidase subunit I mtDNA gene, COI) was amplified for the collected samples using both LepF1/LepRI (Hebert et al. 2003b) and LCO1490/HCO2198 (Folmer et al. 1994) primers. Sequences were edited with Sequencher 4.0.5 (Gene Codes Corp.) and aligned manually based on their translated amino acids. All of the COI sequences generated are deposited in GenBank (accession numbers GU715182-288, HM420734-5, HM434309-544, HM882254, HQ200960-201008, HQ201239-54). All sequences and their specimen information are available in the project file “Parasitoid Wasps (Braconidae: Doryctinae) of Chamela–Cuixmala Biosphere Reserve” (ASDORproject) in the projects section of the Barcode of Life Data Systems (www.barcodinglife.com)

### Species boundaries

Species boundaries were established following a 2% divergence criterion (Hebert et al. 2003a). This criterion has been shown to represent a fast, generally reliable tool for exploring species richness in different animal taxa (Ratnasingham and Hebert 2013). This established genetic distance is based on the assumption that COI divergences usually do not exceed a 2% divergence within a recognized species, whereas different species generally show a higher divergence (Hebert et al. 2003a). Sequences divergence of the sequenced specimens were obtained using the K2P distance model (Kimura 1980). A neighbourjoining (NJ) tree and an accumulation curve were reconstructed as implemented in the BOLD system (Barcode of Life Data System, www.boldsystems.org). BINs were obtained for each specimen from the BOLD system, and this was employed to establish the number of barcoding species.

## Data resources

ASDOR Project link

Search as: ASDOR

http://www.boldsystems.org/index.php/Public_BINSearch?searchtype=records

GenBank link

Search as: Nucleotide + accession number

http://www.ncbi.nlm.nih.gov/genbank/

## Checklists

### Checklist of described doryctine species from the Chamela Biological Station

#### Allorhogas coccolobae

Martínez and Zaldívar-Riverón 2013

http://www.scielo.org.mx/scielo.php?script=sci_arttext&pid=S1870-34532013000100008

##### Materials

- **Type status:** Paratype. **Occurrence:** catalogNumber: ASDOR472-10; recordedBy: Zaldívar, Zaragoza, Ibarra; sex: female; **Taxon:** kingdom: Animalia; phylum: Arthropoda; class: Insecta; order: Hymenoptera; family: Braconidae; genus: Allorhogas; specificEpithet: coccolobae; **Location:** country: Mexico; stateProvince: Jalisco; municipality: La Huerta; locality: Chamela Biostation; decimalLatitude: 19.499; decimalLongitude: -105.042; **Event:** eventDate: 02-20-10
- **Type status:** Paratype. **Occurrence:** catalogNumber: ASDOR517-10; recordedBy: Zaldívar; sex: female; **Taxon:** kingdom: Animalia; phylum: Arthropoda; class: Insecta; order: Hymenoptera; family: Braconidae; genus: Allorhogas; specificEpithet: coccolobae; **Location:** country: Mexico; stateProvince: Jalisco; municipality: La Huerta; locality: Chamela Biostation; decimalLatitude: 19.5; decimalLongitude: -105.039; **Event:** eventDate: 02-21-10
- **Type status:** Paratype. **Occurrence:** catalogNumber: ASDOR518-10; recordedBy: Zaldívar; sex: female; **Taxon:** kingdom: Animalia; phylum: Arthropoda; class: Insecta; order: Hymenoptera; family: Braconidae; genus: Allorhogas; specificEpithet: coccolobae; **Location:** country: Mexico; stateProvince: Jalisco; municipality: La Huerta; locality: Chamela Biostation; decimalLatitude: 19.5; decimalLongitude: -105.039; **Event:** eventDate: 02-21-10
- **Type status:** Paratype. **Occurrence:** catalogNumber: ASDOR519-10; recordedBy: Zaldívar; sex: female; **Taxon:** kingdom: Animalia; phylum: Arthropoda; class: Insecta; order: Hymenoptera; family: Braconidae; genus: Allorhogas; specificEpithet: coccolobae; **Location:** country: Mexico; stateProvince: Jalisco; municipality: La Huerta; locality: Chamela Biostation; decimalLatitude: 19.5; decimalLongitude: -105.039; **Event:** eventDate: 02-21-10
- **Type status:** Paratype. **Occurrence:** catalogNumber: ASDOR764-10; recordedBy: Zaldívar, Salinas, Ramos; sex: male; **Taxon:** kingdom: Animalia; phylum: Arthropoda; class: Insecta; order: Hymenoptera; family: Braconidae; genus: Allorhogas; specificEpithet: coccolobae; **Location:** country: Mexico; stateProvince: Jalisco; municipality: La Huerta; locality: Chamela Biostation; decimalLatitude: 19.504; decimalLongitude: -105.035; **Event:** eventDate: 03-28-10
- **Type status:** Holotype. **Occurrence:** catalogNumber: CNIN777; recordedBy: Zaldívar, Zaragoza, Ibarra; sex: female; **Taxon:** kingdom: Animalia; phylum: Arthropoda; class: Insecta; order: Hymenoptera; family: Braconidae; genus: Allorhogas; specificEpithet: coccolobae; **Location:** country: Mexico; stateProvince: Jalisco; municipality: La Huerta; locality: Chamela Biostation; decimalLatitude: 19.499; decimalLongitude: -105.044; **Event:** eventDate: 05-05-11

##### Distribution

Chamela, Jalisco, Mexico

##### Notes

n.sp. described from specimens collected in this study (Martínez and Zaldívar-Riverón 2013)

#### Allorhogas crassifemur

Martínez and Zaldívar-Riverón 2014

http://www.scielo.org.mx/scielo.php?script=sci_arttext&pid=S1870-34532013000100008

##### Materials

- **Type status:** Paratype. **Occurrence:** catalogNumber: ASDOR042-09; recordedBy: Clebsch, Zaldívar, Polaszek; sex: male; **Taxon:** kingdom: Animalia; phylum: Arthropoda; class: Insecta; order: Hymenoptera; family: Braconidae; genus: Allorhogas; specificEpithet: crassifemur; **Location:** country: Mexico; stateProvince: Jalisco; municipality: La Huerta; locality: Chamela Biostation; decimalLatitude: 19.498; decimalLongitude: -105.044; **Event:** eventDate: 06-24-09
- **Type status:** Holotype. **Occurrence:** catalogNumber: ASDOR043-09; recordedBy: Clebsch, Zaldívar, Polaszek; sex: female; **Taxon:** kingdom: Animalia; phylum: Arthropoda; class: Insecta; order: Hymenoptera; family: Braconidae; genus: Allorhogas; specificEpithet: crassifemur; **Location:** country: Mexico; stateProvince: Jalisco; municipality: La Huerta; locality: Chamela Biostation; decimalLatitude: 19.498; decimalLongitude: -105.044; **Event:** eventDate: 06-23-09

##### Distribution

Chamela, Jalisco, Mexico

##### Notes

n.sp. described from specimens collected in this study (Martínez and Zaldívar-Riverón 2013)

#### Allorhogas jaliscoensis

Martínez and Zaldívar-Riverón 2013

http://www.scielo.org.mx/scielo.php?script=sci_arttext&pid=S1870-34532013000100008

##### Materials

- **Type status:** Paratype. **Occurrence:** catalogNumber: ASDOR081-09; recordedBy: Clebsch, Zaldívar, Polaszek; sex: female; **Taxon:** kingdom: Animalia; phylum: Arthropoda; class: Insecta; order: Hymenoptera; family: Braconidae; genus: Allorhogas; specificEpithet: jaliscoensis; **Location:** country: Mexico; stateProvince: Jalisco; municipality: La Huerta; locality: Chamela Biostation; decimalLatitude: 19.498; decimalLongitude: -105.044; **Event:** eventDate: 09-24-09
- **Type status:** Paratype. **Occurrence:** catalogNumber: ASDOR350-10; recordedBy: Zaldívar; sex: female; **Taxon:** kingdom: Animalia; phylum: Arthropoda; class: Insecta; order: Hymenoptera; family: Braconidae; genus: Allorhogas; specificEpithet: jaliscoensis; **Location:** country: Mexico; stateProvince: Jalisco; municipality: La Huerta; locality: Chamela Biostation; decimalLatitude: 19.505; decimalLongitude: -105.038; **Event:** eventDate: 09-03-09
- **Type status:** Paratype. **Occurrence:** catalogNumber: ASDOR456-10; recordedBy: Zaldívar; sex: female; **Taxon:** kingdom: Animalia; phylum: Arthropoda; class: Insecta; order: Hymenoptera; family: Braconidae; genus: Allorhogas; specificEpithet: jaliscoensis; **Location:** country: Mexico; stateProvince: Jalisco; municipality: La Huerta; locality: Chamela Biostation; decimalLatitude: 19.504; decimalLongitude: -105.038; **Event:** eventDate: 11-20-09
- **Type status:** Holotype. **Occurrence:** catalogNumber: ASDOR457-10; recordedBy: Zaldívar; sex: female; **Taxon:** kingdom: Animalia; phylum: Arthropoda; class: Insecta; order: Hymenoptera; family: Braconidae; genus: Allorhogas; specificEpithet: jaliscoensis; **Location:** country: Mexico; stateProvince: Jalisco; municipality: La Huerta; locality: Chamela Biostation; decimalLatitude: 19.504; decimalLongitude: -105.038; **Event:** eventDate: 11-20-09

##### Distribution

Chamela, Jalisco, Mexico

##### Notes

n.sp. described from specimens collected in this study (Martínez and Zaldívar-Riverón 2013)

#### Allorhogas marshi

Martínez and Zaldívar-Riverón 2013

http://www.scielo.org.mx/scielo.php?script=sci_arttext&pid=S1870-34532013000100008

##### Materials

- **Type status:** Paratype. **Occurrence:** catalogNumber: ASDOR758-10; recordedBy: Zaldívar; sex: female; **Taxon:** kingdom: Animalia; phylum: Arthropoda; class: Insecta; order: Hymenoptera; family: Braconidae; genus: Allorhogas; specificEpithet: marshi; **Location:** country: Mexico; stateProvince: Jalisco; municipality: La Huerta; locality: Chamela Biostation; decimalLatitude: 19.499; decimalLongitude: -105.042; **Event:** eventDate: 02-25-10
- **Type status:** Paratype. **Occurrence:** catalogNumber: ASDOR759-10; recordedBy: Zaldívar; sex: female; **Taxon:** kingdom: Animalia; phylum: Arthropoda; class: Insecta; order: Hymenoptera; family: Braconidae; genus: Allorhogas; specificEpithet: marshi; **Location:** country: Mexico; stateProvince: Jalisco; municipality: La Huerta; locality: Chamela Biostation; decimalLatitude: 19.499; decimalLongitude: -105.042; **Event:** eventDate: 02-25-10
- **Type status:** Paratype. **Occurrence:** catalogNumber: ASDOR760-10; recordedBy: Zaldívar; sex: female; **Taxon:** kingdom: Animalia; phylum: Arthropoda; class: Insecta; order: Hymenoptera; family: Braconidae; genus: Allorhogas; specificEpithet: marshi; **Location:** country: Mexico; stateProvince: Jalisco; municipality: La Huerta; locality: Chamela Biostation; decimalLatitude: 19.499; decimalLongitude: -105.042; **Event:** eventDate: 02-25-10
- **Type status:** Paratype. **Occurrence:** catalogNumber: ASDOR762-10; recordedBy: Zaldívar, Salinas, Ramos; sex: female; **Taxon:** kingdom: Animalia; phylum: Arthropoda; class: Insecta; order: Hymenoptera; family: Braconidae; genus: Allorhogas; specificEpithet: marshi; **Location:** country: Mexico; stateProvince: Jalisco; municipality: La Huerta; locality: Chamela Biostation; decimalLatitude: 19.496; decimalLongitude: -105.039; **Event:** eventDate: 03-28-10
- **Type status:** Paratype. **Occurrence:** catalogNumber: ASDOR763-10; recordedBy: Zaldívar, Salinas, Ramos; sex: female; **Taxon:** kingdom: Animalia; phylum: Arthropoda; class: Insecta; order: Hymenoptera; family: Braconidae; genus: Allorhogas; specificEpithet: marshi; **Location:** country: Mexico; stateProvince: Jalisco; municipality: La Huerta; locality: Chamela Biostation; decimalLatitude: 19.496; decimalLongitude: -105.039; **Event:** eventDate: 03-28-10
- **Type status:** Paratype. **Occurrence:** catalogNumber: ASDOR776-10; recordedBy: Zaldívar; sex: female; **Taxon:** kingdom: Animalia; phylum: Arthropoda; class: Insecta; order: Hymenoptera; family: Braconidae; genus: Allorhogas; specificEpithet: marshi; **Location:** country: Mexico; stateProvince: Jalisco; municipality: La Huerta; locality: Chamela Biostation; decimalLatitude: 19.499; decimalLongitude: -105.042; **Event:** eventDate: 02-25-10
- **Type status:** Holotype. **Occurrence:** catalogNumber: ASDOR777-10; recordedBy: Zaldívar; sex: female; **Taxon:** kingdom: Animalia; phylum: Arthropoda; class: Insecta; order: Hymenoptera; family: Braconidae; genus: Allorhogas; specificEpithet: marshi; **Location:** country: Mexico; stateProvince: Jalisco; municipality: La Huerta; locality: Chamela Biostation; decimalLatitude: 19.499; decimalLongitude: -105.042; **Event:** eventDate: 02-25-10

##### Distribution

Chamela, Jalisco, Mexico

##### Notes

n.sp. described from specimens collected in this study (Martínez and Zaldívar-Riverón 2013)

#### Allorhogas parvus

Martínez and Zaldívar-Riverón 2013

http://www.scielo.org.mx/scielo.php?script=sci_arttext&pid=S1870-34532013000100008

##### Materials

- **Type status:** Paratype. **Occurrence:** catalogNumber: ASDOR458-10; recordedBy: Zaldívar; sex: female; **Taxon:** kingdom: Animalia; phylum: Arthropoda; class: Insecta; order: Hymenoptera; family: Braconidae; genus: Allorhogas; specificEpithet: parvus; **Location:** country: Mexico; stateProvince: Jalisco; municipality: La Huerta; locality: Chamela Biostation; decimalLatitude: 19.499; decimalLongitude: -105.038; **Event:** eventDate: 11-21-09
- **Type status:** Paratype. **Occurrence:** catalogNumber: ASDOR470-10; recordedBy: Zaldívar; sex: female; **Taxon:** kingdom: Animalia; phylum: Arthropoda; class: Insecta; order: Hymenoptera; family: Braconidae; genus: Allorhogas; specificEpithet: parvus; **Location:** country: Mexico; stateProvince: Jalisco; municipality: La Huerta; locality: Chamela Biostation; decimalLatitude: 19.499; decimalLongitude: -105.042; **Event:** eventDate: 02-20-10
- **Type status:** Holotype. **Occurrence:** catalogNumber: ASDOR778-10; recordedBy: Zaldívar, Salinas, Ramos; sex: female; **Taxon:** kingdom: Animalia; phylum: Arthropoda; class: Insecta; order: Hymenoptera; family: Braconidae; genus: Allorhogas; specificEpithet: parvus; **Location:** country: Mexico; stateProvince: Jalisco; municipality: La Huerta; locality: Chamela Biostation; decimalLatitude: 19.499; decimalLongitude: -105.042; **Event:** eventDate: 02-25-10

##### Distribution

Chamela, Jalisco, Mexico

##### Notes

n.sp. described from specimens collected in this study (Martínez and Zaldívar-Riverón 2013)

#### Allorhogas scotti

Martínez and Zaldívar-Riverón 2013

http://www.scielo.org.mx/scielo.php?script=sci_arttext&pid=S1870-34532013000100008

##### Materials

- **Type status:** Paratype. **Occurrence:** catalogNumber: ASDOR321-10; recordedBy: Clebsch, Zaldívar; sex: female; **Taxon:** kingdom: Animalia; phylum: Arthropoda; class: Insecta; order: Hymenoptera; family: Braconidae; genus: Allorhogas; specificEpithet: scotti; **Location:** country: Mexico; stateProvince: Jalisco; municipality: La Huerta; locality: Chamela Biostation; decimalLatitude: 19.429; decimalLongitude: -104.98; **Event:** eventDate: 09-05-09
- **Type status:** Paratype. **Occurrence:** catalogNumber: ASDOR488-10; recordedBy: Zaldívar; sex: female; **Taxon:** kingdom: Animalia; phylum: Arthropoda; class: Insecta; order: Hymenoptera; family: Braconidae; genus: Allorhogas; specificEpithet: scotti; **Location:** country: Mexico; stateProvince: Jalisco; municipality: La Huerta; locality: Chamela Biostation; decimalLatitude: 19.499; decimalLongitude: -105.042; **Event:** eventDate: 02-20-10
- **Type status:** Paratype. **Occurrence:** catalogNumber: ASDOR607-10; recordedBy: Zaldívar; sex: female; **Taxon:** kingdom: Animalia; phylum: Arthropoda; class: Insecta; order: Hymenoptera; family: Braconidae; genus: Allorhogas; specificEpithet: scotti; **Location:** country: Mexico; stateProvince: Jalisco; municipality: La Huerta; locality: Chamela Biostation; decimalLatitude: 19.505; decimalLongitude: -105.038; **Event:** eventDate: 02-23-10
- **Type status:** Paratype. **Occurrence:** catalogNumber: ASDOR745-10; recordedBy: Zaldívar, Salinas, Ramos; sex: female; **Taxon:** kingdom: Animalia; phylum: Arthropoda; class: Insecta; order: Hymenoptera; family: Braconidae; genus: Allorhogas; specificEpithet: scotti; **Location:** country: Mexico; stateProvince: Jalisco; municipality: La Huerta; locality: Chamela Biostation; decimalLatitude: 19.496; decimalLongitude: -105.039; **Event:** eventDate: 03-28-10
- **Type status:** Holotype. **Occurrence:** catalogNumber: ASDOR767-10; recordedBy: Zaldívar, Salinas, Ramos; sex: female; **Taxon:** kingdom: Animalia; phylum: Arthropoda; class: Insecta; order: Hymenoptera; family: Braconidae; genus: Allorhogas; specificEpithet: scotti; **Location:** country: Mexico; stateProvince: Jalisco; municipality: La Huerta; locality: Chamela Biostation; decimalLatitude: 19.504; decimalLongitude: -105.035; **Event:** eventDate: 03-28-10
- **Type status:** Paratype. **Occurrence:** catalogNumber: ASDOR768-10; recordedBy: Zaldívar, Salinas, Ramos; sex: male; **Taxon:** kingdom: Animalia; phylum: Arthropoda; class: Insecta; order: Hymenoptera; family: Braconidae; genus: Allorhogas; specificEpithet: scotti; **Location:** country: Mexico; stateProvince: Jalisco; municipality: La Huerta; locality: Chamela Biostation; decimalLatitude: 19.504; decimalLongitude: -105.035; **Event:** eventDate: 03-28-10

##### Distribution

Chamela, Jalisco, Mexico

##### Notes

n.sp. described from specimens collected in this study (Martínez and Zaldívar-Riverón 2013)

#### Ficobolus jaliscoi

Zaldivar-Riverón and Belokobylskij 2014

http://onlinelibrary.wiley.com/doi/10.1111/syen.12078/abstract;jsessionid=0441E95BA6DBCB93BF1257DF4E1A3B0D.f03t03?deniedAccessCustomisedMessage=&userIsAuthenticated=false

##### Materials

- **Type status:** Paratype. **Occurrence:** catalogNumber: ASDOR446-10; recordedBy: Clebsch, Zaldívar, Polaszek; sex: female; **Taxon:** kingdom: Animalia; phylum: Arthropoda; class: Insecta; order: Hymenoptera; family: Braconidae; genus: Ficobolus; specificEpithet: jaliscoi; **Location:** country: Mexico; stateProvince: Jalisco; municipality: La Huerta; locality: Chamela Biostation; decimalLatitude: 19.499; decimalLongitude: -105.038; **Event:** eventDate: 06-25-09
- **Type status:** Holotype. **Occurrence:** catalogNumber: ASDOR447-10; recordedBy: Clebsch, Zaldívar, Polaszek; sex: female; **Taxon:** kingdom: Animalia; phylum: Arthropoda; class: Insecta; order: Hymenoptera; family: Braconidae; genus: Ficobolus; specificEpithet: jaliscoi; **Location:** country: Mexico; stateProvince: Jalisco; municipality: La Huerta; locality: Chamela Biostation; decimalLatitude: 19.499; decimalLongitude: -105.038; **Event:** eventDate: 06-25-09

##### Distribution

Chamela, Jalisco, Mexico

##### Notes

n.sp. described from specimens collected in this study (Zaldívar-Riverón et al. 2014)

#### Heerz ecmahla

Martínez, Zaldívar-Riverón, Ceccarelli and Shaw 2012

http://zookeys.pensoft.net/articles.php?id=2399

##### Materials

- **Type status:** Paratype. **Occurrence:** catalogNumber: ASDOR075-09; recordedBy: Clebsch, Zaldívar, Polaszek; sex: female; **Taxon:** kingdom: Animalia; phylum: Arthropoda; class: Insecta; order: Hymenoptera; family: Braconidae; genus: Heerz; specificEpithet: ecmahla; **Location:** country: Mexico; stateProvince: Jalisco; municipality: La Huerta; locality: Chamela Biostation; decimalLatitude: 19.498; decimalLongitude: -105.044; **Event:** eventDate: 06-24-09
- **Type status:** Holotype. **Occurrence:** catalogNumber: ASDOR076-09; recordedBy: Clebsch, Zaldívar, Polaszek; sex: female; **Taxon:** kingdom: Animalia; phylum: Arthropoda; class: Insecta; order: Hymenoptera; family: Braconidae; genus: Heerz; specificEpithet: ecmahla; **Location:** country: Mexico; stateProvince: Jalisco; municipality: La Huerta; locality: Chamela Biostation; decimalLatitude: 19.498; decimalLongitude: -105.044; **Event:** eventDate: 06-24-09

##### Distribution

Chamela, Jalisco, Mexico

##### Notes

n.sp. described from specimens collected in this study (Zaldivar-Riveron et al. 2012)

#### Heerz macrophthalma

Martínez, Zaldívar-Riverón, Ceccarelli and Shaw 2013

http://zookeys.pensoft.net/articles.php?id=2400

##### Materials

- **Type status:** Paratype. **Occurrence:** catalogNumber: ASDOR551-10; recordedBy: Zaldívar; sex: male; **Taxon:** kingdom: Animalia; phylum: Arthropoda; class: Insecta; order: Hymenoptera; family: Braconidae; genus: Heerz; specificEpithet: macrophthalma; **Location:** country: Mexico; stateProvince: Jalisco; municipality: La Huerta; locality: Chamela Biostation; decimalLatitude: 19.499; decimalLongitude: -105.044; **Event:** eventDate: 02-02-11
- **Type status:** Paratype. **Occurrence:** catalogNumber: ASDOR555-10; recordedBy: Zaldívar; sex: male; **Taxon:** kingdom: Animalia; phylum: Arthropoda; class: Insecta; order: Hymenoptera; family: Braconidae; genus: Heerz; specificEpithet: macrophthalma; **Location:** country: Mexico; stateProvince: Jalisco; municipality: La Huerta; locality: Chamela Biostation; decimalLatitude: 19.499; decimalLongitude: -105.044; **Event:** eventDate: 02-02-11
- **Type status:** Paratype. **Occurrence:** catalogNumber: ASDOR761-10; recordedBy: Zaldívar; sex: female; **Taxon:** kingdom: Animalia; phylum: Arthropoda; class: Insecta; order: Hymenoptera; family: Braconidae; genus: Heerz; specificEpithet: macrophthalma; **Location:** country: Mexico; stateProvince: Jalisco; municipality: La Huerta; locality: Chamela Biostation; decimalLatitude: 19.499; decimalLongitude: -105.042; **Event:** eventDate: 02-25-10
- **Type status:** Holotype. **Occurrence:** catalogNumber: CNIN795; recordedBy: Zaldívar, Zaragoza, Ibarra; sex: female; **Taxon:** kingdom: Animalia; phylum: Arthropoda; class: Insecta; order: Hymenoptera; family: Braconidae; genus: Heerz; specificEpithet: macrophthalma; **Location:** country: Mexico; stateProvince: Jalisco; municipality: La Huerta; locality: Chamela Biostation; decimalLatitude: 19.499; decimalLongitude: -105.044; **Event:** eventDate: 05-05-11

##### Distribution

Chamela, Jalisco, Mexico

##### Notes

n.sp. described from specimens collected in this study (Zaldivar-Riveron et al. 2012)

#### Iare belokobylskiji

Marsh 2002

http://ejournal.narotama.ac.id/files/The%20genus%20Iare%20Barbalho%20and%20Penteado-Dias%20(Hymenoptera%20Braconidae%20Doryctinae)%20in%20Mexico,%20with%20the%20description%20of%20two%20new%20species.pdf

##### Materials

- **Type status:** Other material. **Occurrence:** catalogNumber: ASDOR022-09; recordedBy: Clebsch, Zaldívar, Polaszek; sex: female; **Taxon:** kingdom: Animalia; phylum: Arthropoda; class: Insecta; order: Hymenoptera; family: Braconidae; genus: Iare; specificEpithet: belokobylskiji; **Location:** country: Mexico; stateProvince: Jalisco; municipality: La Huerta; locality: Chamela Biostation; decimalLatitude: 19.498; decimalLongitude: -105.044; **Event:** eventDate: 06-24-09
- **Type status:** Other material. **Occurrence:** catalogNumber: ASDOR023-09; recordedBy: Clebsch, Zaldívar, Polaszek; sex: female; **Taxon:** kingdom: Animalia; phylum: Arthropoda; class: Insecta; order: Hymenoptera; family: Braconidae; genus: Iare; specificEpithet: belokobylskiji; **Location:** country: Mexico; stateProvince: Jalisco; municipality: La Huerta; locality: Chamela Biostation; decimalLatitude: 19.498; decimalLongitude: -105.044; **Event:** eventDate: 06-24-09
- **Type status:** Other material. **Occurrence:** catalogNumber: ASDOR025-09; recordedBy: Clebsch, Zaldívar, Polaszek; sex: female; **Taxon:** kingdom: Animalia; phylum: Arthropoda; class: Insecta; order: Hymenoptera; family: Braconidae; genus: Iare; specificEpithet: belokobylskiji; **Location:** country: Mexico; stateProvince: Jalisco; municipality: La Huerta; locality: Chamela Biostation; decimalLatitude: 19.498; decimalLongitude: -105.044; **Event:** eventDate: 06-24-09
- **Type status:** Other material. **Occurrence:** catalogNumber: ASDOR026-09; recordedBy: Clebsch, Zaldívar, Polaszek; sex: male; **Taxon:** kingdom: Animalia; phylum: Arthropoda; class: Insecta; order: Hymenoptera; family: Braconidae; genus: Iare; specificEpithet: belokobylskiji; **Location:** country: Mexico; stateProvince: Jalisco; municipality: La Huerta; locality: Chamela Biostation; decimalLatitude: 19.498; decimalLongitude: -105.044; **Event:** eventDate: 06-24-09
- **Type status:** Other material. **Occurrence:** catalogNumber: ASDOR029-09; recordedBy: Clebsch, Zaldívar, Polaszek; sex: female; **Taxon:** kingdom: Animalia; phylum: Arthropoda; class: Insecta; order: Hymenoptera; family: Braconidae; genus: Iare; specificEpithet: belokobylskiji; **Location:** country: Mexico; stateProvince: Jalisco; municipality: La Huerta; locality: Chamela Biostation; decimalLatitude: 19.498; decimalLongitude: -105.044; **Event:** eventDate: 06-24-09
- **Type status:** Other material. **Occurrence:** catalogNumber: ASDOR031-09; recordedBy: Clebsch, Zaldívar, Polaszek; sex: female; **Taxon:** kingdom: Animalia; phylum: Arthropoda; class: Insecta; order: Hymenoptera; family: Braconidae; genus: Iare; specificEpithet: belokobylskiji; **Location:** country: Mexico; stateProvince: Jalisco; municipality: La Huerta; locality: Chamela Biostation; decimalLatitude: 19.498; decimalLongitude: -105.044; **Event:** eventDate: 06-24-09
- **Type status:** Other material. **Occurrence:** catalogNumber: ASDOR035-09; recordedBy: Clebsch, Zaldívar, Polaszek; sex: male; **Taxon:** kingdom: Animalia; phylum: Arthropoda; class: Insecta; order: Hymenoptera; family: Braconidae; genus: Iare; specificEpithet: belokobylskiji; **Location:** country: Mexico; stateProvince: Jalisco; municipality: La Huerta; locality: Chamela Biostation; decimalLatitude: 19.498; decimalLongitude: -105.044; **Event:** eventDate: 06-24-09

##### Distribution

Mexico and Costa Rica

#### Iare cheguevarai

Martínez, Zaldívar-Riverón, Ceccarelli and Shaw 2010

http://ejournal.narotama.ac.id/files/The%20genus%20Iare%20Barbalho%20and%20Penteado-Dias%20(Hymenoptera%20Braconidae%20Doryctinae)%20in%20Mexico,%20with%20the%20description%20of%20two%20new%20species.pdf

##### Materials

- **Type status:** Paratype. **Occurrence:** catalogNumber: ASDOR020-09; recordedBy: Clebsch, Zaldívar, Polaszek; sex: male; **Taxon:** kingdom: Animalia; phylum: Arthropoda; class: Insecta; order: Hymenoptera; family: Braconidae; genus: Iare; specificEpithet: cheguevarai; **Location:** country: Mexico; stateProvince: Jalisco; municipality: La Huerta; locality: Chamela Biostation; decimalLatitude: 19.498; decimalLongitude: -105.044; **Event:** eventDate: 06-24-09
- **Type status:** Paratype. **Occurrence:** catalogNumber: ASDOR021-09; recordedBy: Clebsch, Zaldívar, Polaszek; sex: male; **Taxon:** kingdom: Animalia; phylum: Arthropoda; class: Insecta; order: Hymenoptera; family: Braconidae; genus: Iare; specificEpithet: cheguevarai; **Location:** country: Mexico; stateProvince: Jalisco; municipality: La Huerta; locality: Chamela Biostation; decimalLatitude: 19.498; decimalLongitude: -105.044; **Event:** eventDate: 06-24-09
- **Type status:** Paratype. **Occurrence:** catalogNumber: ASDOR027-09; recordedBy: Clebsch, Zaldívar, Polaszek; sex: female; **Taxon:** kingdom: Animalia; phylum: Arthropoda; class: Insecta; order: Hymenoptera; family: Braconidae; genus: Iare; specificEpithet: cheguevarai; **Location:** country: Mexico; stateProvince: Jalisco; municipality: La Huerta; locality: Chamela Biostation; decimalLatitude: 19.498; decimalLongitude: -105.044; **Event:** eventDate: 06-24-09
- **Type status:** Holotype. **Occurrence:** catalogNumber: ASDOR028-09; recordedBy: Clebsch, Zaldívar, Polaszek; sex: female; **Taxon:** kingdom: Animalia; phylum: Arthropoda; class: Insecta; order: Hymenoptera; family: Braconidae; genus: Iare; specificEpithet: cheguevarai; **Location:** country: Mexico; stateProvince: Jalisco; municipality: La Huerta; locality: Chamela Biostation; decimalLatitude: 19.498; decimalLongitude: -105.044; **Event:** eventDate: 06-24-09
- **Type status:** Paratype. **Occurrence:** catalogNumber: ASDOR096-09; recordedBy: Clebsch, Zaldívar, Polaszek; sex: male; **Taxon:** kingdom: Animalia; phylum: Arthropoda; class: Insecta; order: Hymenoptera; family: Braconidae; genus: Iare; specificEpithet: cheguevarai; **Location:** country: Mexico; stateProvince: Jalisco; municipality: La Huerta; locality: Chamela Biostation; decimalLatitude: 19.498; decimalLongitude: -105.044; **Event:** eventDate: 06-24-09

##### Distribution

Chamela, Jalisco, Mexico

##### Notes

n.sp. described from specimens collected in this study (Martínez et al. 2010)

#### Iare mexicanus

Martínez, Zaldívar-Riverón, Ceccarelli and Shaw 2011

http://ejournal.narotama.ac.id/files/The%20genus%20Iare%20Barbalho%20and%20Penteado-Dias%20(Hymenoptera%20Braconidae%20Doryctinae)%20in%20Mexico,%20with%20the%20description%20of%20two%20new%20species.pdf

##### Materials

- **Type status:** Paratype. **Occurrence:** catalogNumber: ASDOR030-09; recordedBy: Clebsch, Zaldívar, Polaszek; sex: female; **Taxon:** kingdom: Animalia; phylum: Arthropoda; class: Insecta; order: Hymenoptera; family: Braconidae; genus: Iare; specificEpithet: mexicanus; **Location:** country: Mexico; stateProvince: Jalisco; municipality: La Huerta; locality: Chamela Biostation; decimalLatitude: 19.498; decimalLongitude: -105.044; **Event:** eventDate: 06-24-09
- **Type status:** Holotype. **Occurrence:** catalogNumber: ASDOR033-09; recordedBy: Clebsch, Zaldívar, Polaszek; sex: female; **Taxon:** kingdom: Animalia; phylum: Arthropoda; class: Insecta; order: Hymenoptera; family: Braconidae; genus: Iare; specificEpithet: mexicanus; **Location:** country: Mexico; stateProvince: Jalisco; municipality: La Huerta; locality: Chamela Biostation; decimalLatitude: 19.498; decimalLongitude: -105.044; **Event:** eventDate: 06-24-09

##### Distribution

Chamela, Jalisco, Mexico

##### Notes

n.sp. described from specimens collected in this study (Martínez et al. 2010)

#### Lissopsius jaliscoensis

Zaldívar-Riverón, Martínez, Ceccarelli and Shaw 2012

http://zookeys.pensoft.net/articles.php?id=2399

##### Materials

- **Type status:** Paratype. **Occurrence:** catalogNumber: CNIN741; recordedBy: Clebsch, Zaldívar, Polaszek; sex: female; **Taxon:** kingdom: Animalia; phylum: Arthropoda; class: Insecta; order: Hymenoptera; family: Braconidae; genus: Lissopsius; specificEpithet: jaliscoensis; **Location:** country: Mexico; stateProvince: Jalisco; municipality: La Huerta; locality: Chamela Biostation; decimalLatitude: 19.499; decimalLongitude: -105.044; **Event:** eventDate: 06-24-09
- **Type status:** Holotype. **Occurrence:** catalogNumber: CNIN798; recordedBy: Clebsch, Zaldívar, Polaszek; sex: female; **Taxon:** kingdom: Animalia; phylum: Arthropoda; class: Insecta; order: Hymenoptera; family: Braconidae; genus: Lissopsius; specificEpithet: jaliscoensis; **Location:** country: Mexico; stateProvince: Jalisco; municipality: La Huerta; locality: Chamela Biostation; decimalLatitude: 19.499; decimalLongitude: -105.044; **Event:** eventDate: 06-27-09
- **Type status:** Paratype. **Occurrence:** catalogNumber: CNIN798; recordedBy: Clebsch, Zaldívar, Polaszek; sex: female; **Taxon:** kingdom: Animalia; phylum: Arthropoda; class: Insecta; order: Hymenoptera; family: Braconidae; genus: Lissopsius; specificEpithet: jaliscoensis; **Location:** country: Mexico; stateProvince: Jalisco; municipality: La Huerta; locality: Chamela Biostation; decimalLatitude: 19.499; decimalLongitude: -105.044; **Event:** eventDate: 06-24-09
- **Type status:** Paratype. **Occurrence:** catalogNumber: CNIN799; recordedBy: Clebsch, Zaldívar, Polaszek; sex: female; **Taxon:** kingdom: Animalia; phylum: Arthropoda; class: Insecta; order: Hymenoptera; family: Braconidae; genus: Lissopsius; specificEpithet: jaliscoensis; **Location:** country: Mexico; stateProvince: Jalisco; municipality: La Huerta; locality: Chamela Biostation; decimalLatitude: 19.499; decimalLongitude: -105.044; **Event:** eventDate: 06-24-09
- **Type status:** Paratype. **Occurrence:** catalogNumber: CNIN800; recordedBy: Clebsch, Zaldívar, Polaszek; sex: male; **Taxon:** kingdom: Animalia; phylum: Arthropoda; class: Insecta; order: Hymenoptera; family: Braconidae; genus: Lissopsius; specificEpithet: jaliscoensis; **Location:** country: Mexico; stateProvince: Jalisco; municipality: La Huerta; locality: Chamela Biostation; decimalLatitude: 19.499; decimalLongitude: -105.044; **Event:** eventDate: 06-24-09

##### Distribution

Chamela, Jalisco, Mexico

##### Notes

n.sp. described from specimens collected in this study (Zaldivar-Riveron et al. 2012)

#### Lissopsius pacificus

Zaldívar-Riverón, Martínez, ceccarelli and Shaw 2013

http://zookeys.pensoft.net/articles.php?id=2399

##### Materials

- **Type status:** Paratype. **Occurrence:** catalogNumber: CNIN739; recordedBy: Clebsch, Zaldívar, Polaszek; sex: female; **Taxon:** kingdom: Animalia; phylum: Arthropoda; class: Insecta; order: Hymenoptera; family: Braconidae; genus: Lissopsius; specificEpithet: pacificus; **Location:** country: Mexico; stateProvince: Jalisco; municipality: La Huerta; locality: Chamela Biostation; decimalLatitude: 19.499; decimalLongitude: -105.044; **Event:** eventDate: 05-05-11
- **Type status:** Holotype. **Occurrence:** catalogNumber: CNIN740; recordedBy: Clebsch, Zaldívar, Polaszek; sex: female; **Taxon:** kingdom: Animalia; phylum: Arthropoda; class: Insecta; order: Hymenoptera; family: Braconidae; genus: Lissopsius; specificEpithet: pacificus; **Location:** country: Mexico; stateProvince: Jalisco; municipality: La Huerta; locality: Chamela Biostation; decimalLatitude: 19.499; decimalLongitude: -105.044; **Event:** eventDate: 06-26-09
- **Type status:** Paratype. **Occurrence:** catalogNumber: CNIN742; recordedBy: Clebsch, Zaldívar, Polaszek; sex: female; **Taxon:** kingdom: Animalia; phylum: Arthropoda; class: Insecta; order: Hymenoptera; family: Braconidae; genus: Lissopsius; specificEpithet: pacificus; **Location:** country: Mexico; stateProvince: Jalisco; municipality: La Huerta; locality: Chamela Biostation; decimalLatitude: 19.499; decimalLongitude: -105.044; **Event:** eventDate: 05-05-11
- **Type status:** Paratype. **Occurrence:** catalogNumber: CNIN743; recordedBy: Clebsch, Zaldívar, Polaszek; sex: female; **Taxon:** kingdom: Animalia; phylum: Arthropoda; class: Insecta; order: Hymenoptera; family: Braconidae; genus: Lissopsius; specificEpithet: pacificus; **Location:** country: Mexico; stateProvince: Jalisco; municipality: La Huerta; locality: Chamela Biostation; decimalLatitude: 19.499; decimalLongitude: -105.044; **Event:** eventDate: 05-05-11

##### Distribution

Chamela, Jalisco, Mexico

##### Notes

n.sp. described from specimens collected in this study (Zaldivar-Riveron et al. 2012)

#### Monarea fridae

Belokobylskij, Zaldivar-Riverón, Coronado-Blanco 2014

http://dx.doi.org/10.11646/zootaxa.3795.4.2

##### Materials

- **Type status:** Holotype. **Occurrence:** recordedBy: Toledo; sex: female; **Taxon:** kingdom: Animalia; phylum: Arthropoda; class: Insecta; order: Hymenoptera; family: Braconidae; genus: Monarea; specificEpithet: fridae; **Location:** country: Mexico; stateProvince: Morelos; municipality: Tepalcingo; locality: El Limón; decimalLatitude: 12.52; decimalLongitude: -98.94; **Event:** eventDate: 10-13-12
- **Type status:** Paratype. **Occurrence:** recordedBy: Toledo, Hinterholzer, Martínez; sex: male; **Taxon:** kingdom: Animalia; phylum: Arthropoda; class: Insecta; order: Hymenoptera; family: Braconidae; genus: Monarea; specificEpithet: fridae; **Location:** country: Mexico; stateProvince: Puebla; municipality: Jolalpan; locality: Rancho el Salado; decimalLatitude: 18.33; decimalLongitude: -98.98; **Event:** eventDate: 10-07-10
- **Type status:** Paratype. **Occurrence:** recordedBy: Toledo; sex: male; **Taxon:** kingdom: Animalia; phylum: Arthropoda; class: Insecta; order: Hymenoptera; family: Braconidae; genus: Monarea; specificEpithet: fridae; **Location:** country: Mexico; stateProvince: Morelos; municipality: Tlaquiltenango; locality: Santiopa; decimalLatitude: 18.44; decimalLongitude: -98.95; **Event:** eventDate: 07-06-13
- **Type status:** Paratype. **Occurrence:** recordedBy: Toledo; sex: male; **Taxon:** kingdom: Animalia; phylum: Arthropoda; class: Insecta; order: Hymenoptera; family: Braconidae; genus: Monarea; specificEpithet: fridae; **Location:** country: Mexico; stateProvince: Jalisco; municipality: La Huerta; locality: Chamela Biostation; decimalLatitude: 19.49; decimalLongitude: -105.044; **Event:** eventDate: 06-06-11

##### Distribution

Central Mexico and Jalisco

##### Notes

n.sp. described from specimens collected in this study (Belokobyskij et al. 2014)

#### Neoheterospilus chamelae

Marínez and Zaldívar-Riverón 2010

http://www.conabio.gob.mx/institucion/proyectos/resultados/HB033_Neoheterospilus%202010.pdf

##### Materials

- **Type status:** Holotype. **Occurrence:** catalogNumber: ASDOR053-09; recordedBy: Clebsch, Zaldívar, Polaszek; sex: female; **Taxon:** kingdom: Animalia; phylum: Arthropoda; class: Insecta; order: Hymenoptera; family: Braconidae; genus: Neoheterospilus; specificEpithet: chamelae; **Location:** country: Mexico; stateProvince: Jalisco; municipality: La Huerta; locality: Chamela Biostation; decimalLatitude: 19.498; decimalLongitude: -105.044
- **Type status:** Paratype. **Occurrence:** catalogNumber: ASDOR054-09; recordedBy: Zaldívar; sex: female; **Taxon:** kingdom: Animalia; phylum: Arthropoda; class: Insecta; order: Hymenoptera; family: Braconidae; genus: Neoheterospilus; specificEpithet: chamelae; **Location:** country: Mexico; stateProvince: Jalisco; municipality: La Huerta; locality: Chamela Biostation; decimalLatitude: 19.498; decimalLongitude: -105.044
- **Type status:** Paratype. **Occurrence:** catalogNumber: ASDOR095-09; recordedBy: Zaldívar; sex: female; **Taxon:** kingdom: Animalia; phylum: Arthropoda; class: Insecta; order: Hymenoptera; family: Braconidae; genus: Neoheterospilus; specificEpithet: chamelae; **Location:** country: Mexico; stateProvince: Jalisco; municipality: La Huerta; locality: Chamela Biostation; decimalLatitude: 19.499; decimalLongitude: -105.038
- **Type status:** Other material. **Occurrence:** catalogNumber: ASDOR108-09; recordedBy: Zaldívar; sex: female; **Taxon:** kingdom: Animalia; phylum: Arthropoda; class: Insecta; order: Hymenoptera; family: Braconidae; genus: Neoheterospilus; specificEpithet: chamelae; **Location:** country: Mexico; stateProvince: Jalisco; municipality: La Huerta; locality: Chamela Biostation; decimalLatitude: 19.499; decimalLongitude: -105.038
- **Type status:** Other material. **Occurrence:** catalogNumber: ASDOR148-09; recordedBy: Zaldívar; sex: female; **Taxon:** kingdom: Animalia; phylum: Arthropoda; class: Insecta; order: Hymenoptera; family: Braconidae; genus: Neoheterospilus; specificEpithet: chamelae; **Location:** country: Mexico; stateProvince: Jalisco; municipality: La Huerta; locality: Chamela Biostation; decimalLatitude: 19.497; decimalLongitude: -105.038
- **Type status:** Paratype. **Occurrence:** catalogNumber: ASDOR202-10; recordedBy: Zaldívar; sex: female; **Taxon:** kingdom: Animalia; phylum: Arthropoda; class: Insecta; order: Hymenoptera; family: Braconidae; genus: Neoheterospilus; specificEpithet: chamelae; **Location:** country: Mexico; stateProvince: Jalisco; municipality: La Huerta; locality: Chamela Biostation; decimalLatitude: 19.499; decimalLongitude: -105.038
- **Type status:** Paratype. **Occurrence:** catalogNumber: ASDOR216-10; recordedBy: Zaldívar; sex: male; **Taxon:** kingdom: Animalia; phylum: Arthropoda; class: Insecta; order: Hymenoptera; family: Braconidae; genus: Neoheterospilus; specificEpithet: chamelae; **Location:** country: Mexico; stateProvince: Jalisco; municipality: La Huerta; locality: Chamela Biostation; decimalLatitude: 19.498; decimalLongitude: -105.045
- **Type status:** Paratype. **Occurrence:** catalogNumber: ASDOR343-10; recordedBy: Zaldívar; sex: male; **Taxon:** kingdom: Animalia; phylum: Arthropoda; class: Insecta; order: Hymenoptera; family: Braconidae; genus: Neoheterospilus; specificEpithet: chamelae; **Location:** country: Mexico; stateProvince: Jalisco; municipality: La Huerta; locality: Chamela Biostation; decimalLatitude: 19.429; decimalLongitude: -104.98
- **Type status:** Paratype. **Occurrence:** catalogNumber: ASDOR392-10; recordedBy: Zaldívar; sex: male; **Taxon:** kingdom: Animalia; phylum: Arthropoda; class: Insecta; order: Hymenoptera; family: Braconidae; genus: Neoheterospilus; specificEpithet: chamelae; **Location:** country: Mexico; stateProvince: Jalisco; municipality: La Huerta; locality: Chamela Biostation; decimalLatitude: 19.499; decimalLongitude: -105.044
- **Type status:** Paratype. **Occurrence:** catalogNumber: ASDOR418-10; recordedBy: Zaldívar; sex: male; **Taxon:** kingdom: Animalia; phylum: Arthropoda; class: Insecta; order: Hymenoptera; family: Braconidae; genus: Neoheterospilus; specificEpithet: chamelae; **Location:** country: Mexico; stateProvince: Jalisco; municipality: La Huerta; locality: Chamela Biostation; decimalLatitude: 19.499; decimalLongitude: -105.038
- **Type status:** Paratype. **Occurrence:** catalogNumber: ASDOR473-10; recordedBy: Zaldívar; sex: female; **Taxon:** kingdom: Animalia; phylum: Arthropoda; class: Insecta; order: Hymenoptera; family: Braconidae; genus: Neoheterospilus; specificEpithet: chamelae; **Location:** country: Mexico; stateProvince: Jalisco; municipality: La Huerta; locality: Chamela Biostation; decimalLatitude: 19.499; decimalLongitude: -105.042
- **Type status:** Paratype. **Occurrence:** catalogNumber: ASDOR482-10; recordedBy: Zaldívar; sex: female; **Taxon:** kingdom: Animalia; phylum: Arthropoda; class: Insecta; order: Hymenoptera; family: Braconidae; genus: Neoheterospilus; specificEpithet: chamelae; **Location:** country: Mexico; stateProvince: Jalisco; municipality: La Huerta; locality: Chamela Biostation; decimalLatitude: 19.499; decimalLongitude: -105.042
- **Type status:** Paratype. **Occurrence:** catalogNumber: ASDOR492-10; recordedBy: Zaldívar; sex: male; **Taxon:** kingdom: Animalia; phylum: Arthropoda; class: Insecta; order: Hymenoptera; family: Braconidae; genus: Neoheterospilus; specificEpithet: chamelae; **Location:** country: Mexico; stateProvince: Jalisco; municipality: La Huerta; locality: Chamela Biostation; decimalLatitude: 19.499; decimalLongitude: -105.042
- **Type status:** Paratype. **Occurrence:** catalogNumber: ASDOR549-10; recordedBy: Zaldívar; sex: male; **Taxon:** kingdom: Animalia; phylum: Arthropoda; class: Insecta; order: Hymenoptera; family: Braconidae; genus: Neoheterospilus; specificEpithet: chamelae; **Location:** country: Mexico; stateProvince: Jalisco; municipality: La Huerta; locality: Chamela Biostation; decimalLatitude: 19.509; decimalLongitude: -105.037
- **Type status:** Paratype. **Occurrence:** catalogNumber: ASDOR550-10; recordedBy: Zaldívar; sex: male; **Taxon:** kingdom: Animalia; phylum: Arthropoda; class: Insecta; order: Hymenoptera; family: Braconidae; genus: Neoheterospilus; specificEpithet: chamelae; **Location:** country: Mexico; stateProvince: Jalisco; municipality: La Huerta; locality: Chamela Biostation; decimalLatitude: 19.509; decimalLongitude: -105.037
- **Type status:** Paratype. **Occurrence:** catalogNumber: ASDOR565-10; recordedBy: Zaldívar; sex: female; **Taxon:** kingdom: Animalia; phylum: Arthropoda; class: Insecta; order: Hymenoptera; family: Braconidae; genus: Neoheterospilus; specificEpithet: chamelae; **Location:** country: Mexico; stateProvince: Jalisco; municipality: La Huerta; locality: Chamela Biostation; decimalLatitude: 19.499; decimalLongitude: -105.044
- **Type status:** Paratype. **Occurrence:** catalogNumber: ASDOR566-10; recordedBy: Zaldívar; sex: female; **Taxon:** kingdom: Animalia; phylum: Arthropoda; class: Insecta; order: Hymenoptera; family: Braconidae; genus: Neoheterospilus; specificEpithet: chamelae; **Location:** country: Mexico; stateProvince: Jalisco; municipality: La Huerta; locality: Chamela Biostation; decimalLatitude: 19.499; decimalLongitude: -105.044
- **Type status:** Paratype. **Occurrence:** catalogNumber: ASDOR567-10; recordedBy: Zaldívar; sex: male; **Taxon:** kingdom: Animalia; phylum: Arthropoda; class: Insecta; order: Hymenoptera; family: Braconidae; genus: Neoheterospilus; specificEpithet: chamelae; **Location:** country: Mexico; stateProvince: Jalisco; municipality: La Huerta; locality: Chamela Biostation; decimalLatitude: 19.499; decimalLongitude: -105.044
- **Type status:** Paratype. **Occurrence:** catalogNumber: ASDOR601-10; recordedBy: Zaldívar; sex: male; **Taxon:** kingdom: Animalia; phylum: Arthropoda; class: Insecta; order: Hymenoptera; family: Braconidae; genus: Neoheterospilus; specificEpithet: chamelae; **Location:** country: Mexico; stateProvince: Jalisco; municipality: La Huerta; locality: Chamela Biostation; decimalLatitude: 19.505; decimalLongitude: -105.038
- **Type status:** Paratype. **Occurrence:** catalogNumber: ASDOR625-10; recordedBy: Zaldívar; sex: male; **Taxon:** kingdom: Animalia; phylum: Arthropoda; class: Insecta; order: Hymenoptera; family: Braconidae; genus: Neoheterospilus; specificEpithet: chamelae; **Location:** country: Mexico; stateProvince: Jalisco; municipality: La Huerta; locality: Chamela Biostation; decimalLatitude: 19.505; decimalLongitude: -105.038
- **Type status:** Paratype. **Occurrence:** catalogNumber: ASDOR627-10; recordedBy: Zaldívar; sex: male; **Taxon:** kingdom: Animalia; phylum: Arthropoda; class: Insecta; order: Hymenoptera; family: Braconidae; genus: Neoheterospilus; specificEpithet: chamelae; **Location:** country: Mexico; stateProvince: Jalisco; municipality: La Huerta; locality: Chamela Biostation; decimalLatitude: 19.505; decimalLongitude: -105.038
- **Type status:** Paratype. **Occurrence:** catalogNumber: ASDOR632-10; recordedBy: Zaldívar; sex: female; **Taxon:** kingdom: Animalia; phylum: Arthropoda; class: Insecta; order: Hymenoptera; family: Braconidae; genus: Neoheterospilus; specificEpithet: chamelae; **Location:** country: Mexico; stateProvince: Jalisco; municipality: La Huerta; locality: Chamela Biostation; decimalLatitude: 19.505; decimalLongitude: -105.038
- **Type status:** Paratype. **Occurrence:** catalogNumber: ASDOR633-10; recordedBy: Zaldívar; sex: female; **Taxon:** kingdom: Animalia; phylum: Arthropoda; class: Insecta; order: Hymenoptera; family: Braconidae; genus: Neoheterospilus; specificEpithet: chamelae; **Location:** country: Mexico; stateProvince: Jalisco; municipality: La Huerta; locality: Chamela Biostation; decimalLatitude: 19.505; decimalLongitude: -105.038
- **Type status:** Paratype. **Occurrence:** catalogNumber: ASDOR697-10; recordedBy: Zaldívar; sex: male; **Taxon:** kingdom: Animalia; phylum: Arthropoda; class: Insecta; order: Hymenoptera; family: Braconidae; genus: Neoheterospilus; specificEpithet: chamelae; **Location:** country: Mexico; stateProvince: Jalisco; municipality: La Huerta; locality: Chamela Biostation; decimalLatitude: 19.499; decimalLongitude: -105.042
- **Type status:** Other material. **Occurrence:** catalogNumber: ASDOR718-10; recordedBy: Zaldívar; sex: male; **Taxon:** kingdom: Animalia; phylum: Arthropoda; class: Insecta; order: Hymenoptera; family: Braconidae; genus: Neoheterospilus; specificEpithet: chamelae; **Location:** country: Mexico; stateProvince: Jalisco; municipality: La Huerta; locality: Chamela Biostation; decimalLatitude: 19.504; decimalLongitude: -105.035
- **Type status:** Other material. **Occurrence:** catalogNumber: ASDOR737-10; recordedBy: Zaldívar; sex: male; **Taxon:** kingdom: Animalia; phylum: Arthropoda; class: Insecta; order: Hymenoptera; family: Braconidae; genus: Neoheterospilus; specificEpithet: chamelae; **Location:** country: Mexico; stateProvince: Jalisco; municipality: La Huerta; locality: Chamela Biostation; decimalLatitude: 19.496; decimalLongitude: -105.039
- **Type status:** Other material. **Occurrence:** catalogNumber: ASDOR769-10; recordedBy: Zaldívar; sex: female; **Taxon:** kingdom: Animalia; phylum: Arthropoda; class: Insecta; order: Hymenoptera; family: Braconidae; genus: Neoheterospilus; specificEpithet: chamelae; **Location:** country: Mexico; stateProvince: Jalisco; municipality: La Huerta; locality: Chamela Biostation; decimalLatitude: 19.499; decimalLongitude: -105.042
- **Type status:** Other material. **Occurrence:** catalogNumber: ASDOR770-10; recordedBy: Zaldívar; sex: male; **Taxon:** kingdom: Animalia; phylum: Arthropoda; class: Insecta; order: Hymenoptera; family: Braconidae; genus: Neoheterospilus; specificEpithet: chamelae; **Location:** country: Mexico; stateProvince: Jalisco; municipality: La Huerta; locality: Chamela Biostation; decimalLatitude: 19.499; decimalLongitude: -105.042
- **Type status:** Other material. **Occurrence:** catalogNumber: ASDOR772-10; recordedBy: Zaldívar; sex: female; **Taxon:** kingdom: Animalia; phylum: Arthropoda; class: Insecta; order: Hymenoptera; family: Braconidae; genus: Neoheterospilus; specificEpithet: chamelae; **Location:** country: Mexico; stateProvince: Jalisco; municipality: La Huerta; locality: Chamela Biostation; decimalLatitude: 19.5; decimalLongitude: -105.039
- **Type status:** Other material. **Occurrence:** catalogNumber: ASDOR773-10; recordedBy: Zaldívar; sex: female; **Taxon:** kingdom: Animalia; phylum: Arthropoda; class: Insecta; order: Hymenoptera; family: Braconidae; genus: Neoheterospilus; specificEpithet: chamelae; **Location:** country: Mexico; stateProvince: Jalisco; municipality: La Huerta; locality: Chamela Biostation; decimalLatitude: 19.499; decimalLongitude: -105.038
- **Type status:** Other material. **Occurrence:** catalogNumber: ASDOR775-10; recordedBy: Zaldívar; sex: male; **Taxon:** kingdom: Animalia; phylum: Arthropoda; class: Insecta; order: Hymenoptera; family: Braconidae; genus: Neoheterospilus; specificEpithet: chamelae; **Location:** country: Mexico; stateProvince: Jalisco; municipality: La Huerta; locality: Chamela Biostation; decimalLatitude: 19.499; decimalLongitude: -105.038
- **Type status:** Other material. **Occurrence:** catalogNumber: ASDOR789-10; recordedBy: Zaldívar; sex: male; **Taxon:** kingdom: Animalia; phylum: Arthropoda; class: Insecta; order: Hymenoptera; family: Braconidae; genus: Neoheterospilus; specificEpithet: chamelae; **Location:** country: Mexico; stateProvince: Jalisco; municipality: La Huerta; locality: Chamela Biostation; decimalLatitude: 19.499; decimalLongitude: -105.044
- **Type status:** Other material. **Occurrence:** catalogNumber: ASDOR816-10; recordedBy: Zaldívar; sex: male; **Taxon:** kingdom: Animalia; phylum: Arthropoda; class: Insecta; order: Hymenoptera; family: Braconidae; genus: Neoheterospilus; specificEpithet: chamelae; **Location:** country: Mexico; stateProvince: Jalisco; municipality: La Huerta; locality: Chamela Biostation; decimalLatitude: 19.499; decimalLongitude: -105.044
- **Type status:** Other material. **Occurrence:** catalogNumber: ASDOR820-10; recordedBy: Zaldívar; sex: male; **Taxon:** kingdom: Animalia; phylum: Arthropoda; class: Insecta; order: Hymenoptera; family: Braconidae; genus: Neoheterospilus; specificEpithet: chamelae; **Location:** country: Mexico; stateProvince: Jalisco; municipality: La Huerta; locality: Chamela Biostation; decimalLatitude: 19.499; decimalLongitude: -105.044
- **Type status:** Other material. **Occurrence:** catalogNumber: ASDOR822-10; recordedBy: Zaldívar; sex: male; **Taxon:** kingdom: Animalia; phylum: Arthropoda; class: Insecta; order: Hymenoptera; family: Braconidae; genus: Neoheterospilus; specificEpithet: chamelae; **Location:** country: Mexico; stateProvince: Jalisco; municipality: La Huerta; locality: Chamela Biostation; decimalLatitude: 19.49; decimalLongitude: -105.038
- **Type status:** Other material. **Occurrence:** catalogNumber: ASDOR853-10; recordedBy: Zaldívar; sex: female; **Taxon:** kingdom: Animalia; phylum: Arthropoda; class: Insecta; order: Hymenoptera; family: Braconidae; genus: Neoheterospilus; specificEpithet: chamelae; **Location:** country: Mexico; stateProvince: Jalisco; municipality: La Huerta; locality: Chamela Biostation; decimalLatitude: 19.499; decimalLongitude: -105.044

##### Distribution

Chamela, Jalisco, Mexico

##### Notes

n.sp. described from specimens collected in this study (Martínez and Zaldívar-Riverón 2010)

#### Notiospathius crypticus

Reséndiz-Flores, Nunes and Zaldívar Riverón 2014

http://www.ib.unam.mx/m/revista/pdfs/05.-_1581_1.pdf

##### Materials

- **Type status:** Holotype. **Occurrence:** catalogNumber: ASDOR016-09; recordedBy: Clebsch, Zaldívar, Polaszek; sex: female; **Taxon:** kingdom: Animalia; phylum: Arthropoda; class: Insecta; order: Hymenoptera; family: Braconidae; genus: Notiospathius; specificEpithet: crypticus; **Location:** country: Mexico; stateProvince: Jalisco; municipality: La Huerta; locality: Chamela Biostation; decimalLatitude: 19.499; decimalLongitude: -105.038; **Event:** eventDate: 06-25-09
- **Type status:** Paratype. **Occurrence:** catalogNumber: ASDOR017-09; recordedBy: Clebsch, Zaldívar, Polaszek; sex: female; **Taxon:** kingdom: Animalia; phylum: Arthropoda; class: Insecta; order: Hymenoptera; family: Braconidae; genus: Notiospathius; specificEpithet: crypticus; **Location:** country: Mexico; stateProvince: Jalisco; municipality: La Huerta; locality: Chamela Biostation; decimalLatitude: 19.499; decimalLongitude: -105.038; **Event:** eventDate: 06-26-09

##### Distribution

Chamela, Jalisco, Mexico

##### Notes

n.sp. described from specimens collected in this study (Reséndiz-Flores et al. 2014)

#### Notiospathius mariachi

Reséndiz-Flores, Nunes and Zaldívar Riverón 2014

http://www.ib.unam.mx/m/revista/pdfs/05.-_1581_1.pdf

##### Materials

- **Type status:** Paratype. **Occurrence:** catalogNumber: ASDOR018-09; recordedBy: Clebsch, Zaldívar, Polaszek; sex: female; **Taxon:** kingdom: Animalia; phylum: Arthropoda; class: Insecta; order: Hymenoptera; family: Braconidae; genus: Notiospathius; specificEpithet: mariachi; **Location:** country: Mexico; stateProvince: Jalisco; municipality: La Huerta; locality: Chamela Biostation; decimalLatitude: 19.499; decimalLongitude: -105.038; **Event:** eventDate: 06-26-09
- **Type status:** Paratype. **Occurrence:** catalogNumber: ASDOR019-09; recordedBy: Clebsch, Zaldívar, Polaszek; sex: male; **Taxon:** kingdom: Animalia; phylum: Arthropoda; class: Insecta; order: Hymenoptera; family: Braconidae; genus: Notiospathius; specificEpithet: mariachi; **Location:** country: Mexico; stateProvince: Jalisco; municipality: La Huerta; locality: Chamela Biostation; decimalLatitude: 19.499; decimalLongitude: -105.038; **Event:** eventDate: 06-27-09
- **Type status:** Paratype. **Occurrence:** catalogNumber: ASDOR355-10; recordedBy: Clebsch, Zaldívar, Polaszek; sex: female; **Taxon:** kingdom: Animalia; phylum: Arthropoda; class: Insecta; order: Hymenoptera; family: Braconidae; genus: Notiospathius; specificEpithet: mariachi; **Location:** country: Mexico; stateProvince: Jalisco; municipality: La Huerta; locality: Chamela Biostation; decimalLatitude: 19.505; decimalLongitude: -105.038; **Event:** eventDate: 09-04-09
- **Type status:** Paratype. **Occurrence:** catalogNumber: ASDOR357-10; recordedBy: Clebsch, Zaldívar, Polaszek; sex: male; **Taxon:** kingdom: Animalia; phylum: Arthropoda; class: Insecta; order: Hymenoptera; family: Braconidae; genus: Notiospathius; specificEpithet: mariachi; **Location:** country: Mexico; stateProvince: Jalisco; municipality: La Huerta; locality: Chamela Biostation; decimalLatitude: 19.505; decimalLongitude: -105.038; **Event:** eventDate: 09-03-09
- **Type status:** Holotype. **Occurrence:** catalogNumber: ASDOR463-10; recordedBy: Zaldívar; sex: female; **Taxon:** kingdom: Animalia; phylum: Arthropoda; class: Insecta; order: Hymenoptera; family: Braconidae; genus: Notiospathius; specificEpithet: mariachi; **Location:** country: Mexico; stateProvince: Jalisco; municipality: La Huerta; locality: Chamela Biostation; decimalLatitude: 19.504; decimalLongitude: -105.038; **Event:** eventDate: 11-20-09

##### Distribution

Chamela, Jalisco, Mexico

##### Notes

n.sp. described from specimens collected in this study (Reséndiz-Flores et al. 2014)

#### Ondigus cuixmalensis

Zaldívar-Riverón, Martínez, ceccarelli and Shaw 2014

http://zookeys.pensoft.net/articles.php?id=2399

##### Materials

- **Type status:** Holotype. **Occurrence:** catalogNumber: ASDOR464-10; recordedBy: Clebsch, Zaldívar; sex: female; **Taxon:** kingdom: Animalia; phylum: Arthropoda; class: Insecta; order: Hymenoptera; family: Braconidae; genus: Ondigus; specificEpithet: cuixmalensis; **Location:** country: Mexico; stateProvince: Jalisco; municipality: La Huerta; locality: Chamela Biostation; decimalLatitude: 19.419; decimalLongitude: -104.973; **Event:** eventDate: 09-03-09
- **Type status:** Paratype. **Occurrence:** catalogNumber: ASDOR514-10; recordedBy: Zaldívar; sex: male; **Taxon:** kingdom: Animalia; phylum: Arthropoda; class: Insecta; order: Hymenoptera; family: Braconidae; genus: Ondigus; specificEpithet: cuixmalensis; **Location:** country: Mexico; stateProvince: Jalisco; municipality: La Huerta; locality: Chamela Biostation; decimalLatitude: 19.505; decimalLongitude: -105.038; **Event:** eventDate: 02-20-10

##### Distribution

Chamela, Jalisco, Mexico

##### Notes

n.sp. described from specimens collected in this study (Zaldivar-Riveron et al. 2012)

#### Sabinita mexicana

Belokobylskij, Zaldivar-Riverón and Martínez 2014

http://onlinelibrary.wiley.com/doi/10.1111/syen.12078/abstract;jsessionid=0441E95BA6DBCB93BF1257DF4E1A3B0D.f03t03?deniedAccessCustomisedMessage=&userIsAuthenticated=false

##### Materials

- **Type status:** Holotype. **Occurrence:** catalogNumber: ASDOR082-09; recordedBy: Zaldívar; sex: female; **Taxon:** kingdom: Animalia; phylum: Arthropoda; class: Insecta; order: Hymenoptera; family: Braconidae; genus: Sabinita; specificEpithet: mexicana; **Location:** country: Mexico; stateProvince: Jalisco; municipality: La Huerta; locality: Chamela Biostation; decimalLatitude: 19.497; decimalLongitude: -105.038; **Event:** eventDate: 07-02-12

##### Distribution

Chamela, Jalisco, Mexico

##### Notes

n.sp. described from specimens collected in this study (Zaldívar-Riverón et al. 2014)

#### Spathius chamelae

Belokobylskij and Zaldivar-Riverón 2014

http://www.ncbi.nlm.nih.gov/pmc/articles/PMC4137313/pdf/zookeys-427-059.pdf

##### Materials

- **Type status:** Paratype. **Occurrence:** catalogNumber: ASDOR371-10; recordedBy: Clebsch, Zaldívar; sex: male; **Taxon:** kingdom: Animalia; phylum: Arthropoda; class: Insecta; order: Hymenoptera; family: Braconidae; genus: Spathius; specificEpithet: chamelae; **Location:** country: Mexico; stateProvince: Jalisco; municipality: La Huerta; locality: Chamela Biostation; decimalLatitude: 19.505; decimalLongitude: -105.038
- **Type status:** Paratype. **Occurrence:** catalogNumber: ASDOR372-10; recordedBy: Clebsch, Zaldívar; sex: female; **Taxon:** kingdom: Animalia; phylum: Arthropoda; class: Insecta; order: Hymenoptera; family: Braconidae; genus: Spathius; specificEpithet: chamelae; **Location:** country: Mexico; stateProvince: Jalisco; municipality: La Huerta; locality: Chamela Biostation; decimalLatitude: 19.505; decimalLongitude: -105.038
- **Type status:** Paratype. **Occurrence:** catalogNumber: ASDOR373-10; recordedBy: Clebsch, Zaldívar; sex: female; **Taxon:** kingdom: Animalia; phylum: Arthropoda; class: Insecta; order: Hymenoptera; family: Braconidae; genus: Spathius; specificEpithet: chamelae; **Location:** country: Mexico; stateProvince: Jalisco; municipality: La Huerta; locality: Chamela Biostation; decimalLatitude: 19.505; decimalLongitude: -105.038
- **Type status:** Paratype. **Occurrence:** catalogNumber: ASDOR375-10; recordedBy: Clebsch, Zaldívar; sex: male; **Taxon:** kingdom: Animalia; phylum: Arthropoda; class: Insecta; order: Hymenoptera; family: Braconidae; genus: Spathius; specificEpithet: chamelae; **Location:** country: Mexico; stateProvince: Jalisco; municipality: La Huerta; locality: Chamela Biostation; decimalLatitude: 19.505; decimalLongitude: -105.038
- **Type status:** Paratype. **Occurrence:** catalogNumber: ASDOR432-10; recordedBy: Clebsch, Zaldívar; sex: male; **Taxon:** kingdom: Animalia; phylum: Arthropoda; class: Insecta; order: Hymenoptera; family: Braconidae; genus: Spathius; specificEpithet: chamelae; **Location:** country: Mexico; stateProvince: Jalisco; municipality: La Huerta; locality: Chamela Biostation; decimalLatitude: 19.505; decimalLongitude: -105.038
- **Type status:** Holotype. **Occurrence:** catalogNumber: ASDOR433-10; recordedBy: Zaldívar; sex: female; **Taxon:** kingdom: Animalia; phylum: Arthropoda; class: Insecta; order: Hymenoptera; family: Braconidae; genus: Spathius; specificEpithet: chamelae; **Location:** country: Mexico; stateProvince: Jalisco; municipality: La Huerta; locality: Chamela Biostation; decimalLatitude: 19.505; decimalLongitude: -105.038

##### Distribution

Chamela, Jalisco, Mexico

##### Notes

n.sp. described from specimens collected in this study (Belokobylskij and Zaldivar-Riveron 2014)

#### Whitfieldiellus variegata

Marsh 1993

http://doryctinaekey.myspecies.info/file/339

##### Materials

- **Type status:** Other material. **Occurrence:** catalogNumber: ASDOR001-09; recordedBy: Clebsch, Zaldívar, Polaszek; sex: female; **Taxon:** kingdom: Animalia; phylum: Arthropoda; class: Insecta; order: Hymenoptera; family: Braconidae; genus: Whitfieldiellus; specificEpithet: variegata; **Location:** country: Mexico; stateProvince: Jalisco; municipality: La Huerta; locality: Chamela Biostation; decimalLatitude: 19.498; decimalLongitude: -105.044; **Event:** eventDate: 06-24-09

##### Distribution

Mexico, Guatemala, Panama and Costa Rica

## Analysis

### Species boundaries

A total of 961 specimens were collected during the ten field trips, of which 883 COI sequences were generated. Fifteen of these sequences had a length lower than 500 bp and thus did not receive a BIN, though they were included in the analysis. The sequences generated belonged to 289 barcoding species and 30 identified genera, though four barcoding species could not be assigned to any genus (Table 2). *Heterospilus* Haliday was the most speciose genera with 170 barcoding species, followed by *Ecphylus* Förster (19 spp.), *Allorhogas* Gahan (15 spp.) and *Callihormius* Ashmead (14 spp.). Two species belonging to two additional genera (*Doryctinus* Roman; *Monarea fridae*, Belokobyskij et al. 2014 were foundfound in the entomological collection at the CBS, increasing the diversity of doryctine genera and species present in the region to 33 and 289, respectively.

**Figure 2.**
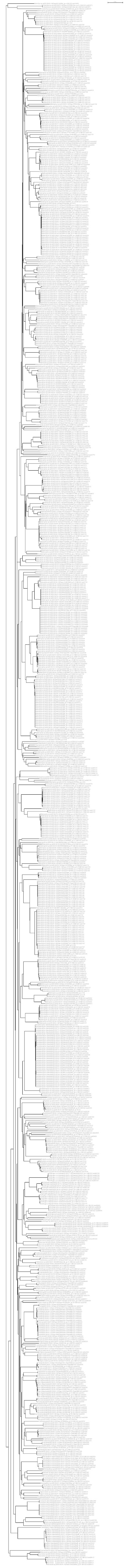
Neighbour-joining tree obtained from BOLD that includes 883 nucleotide sequences belonging to doryctine specimens. The distance model used was the Kimura 2 Parameter, the marker was COI-5P, and the 1st, 2nd and 3rd codon positions were included. The sequences had a lengrh greater than 200bp. See Suppl. material 1.

**Figure 3.**
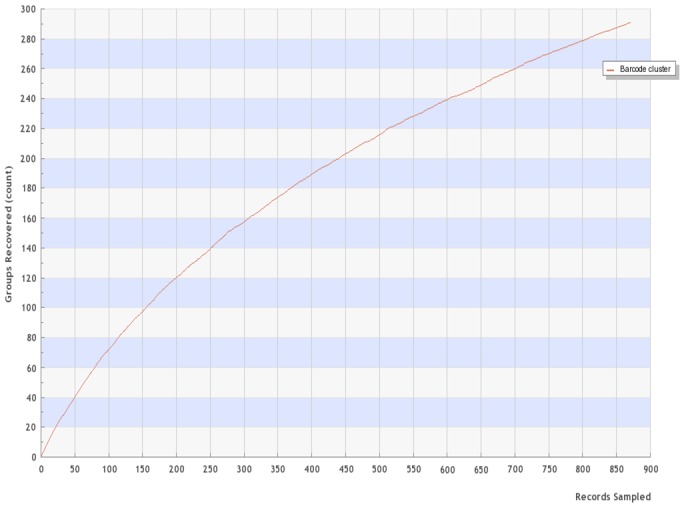
DNA barcoding species accumulation curve for the Doryctinae from the CBS (Suppl. material 1).

The reconstructed NJ tree recovered the megadiverse genus *Heterospilus* as non-monophyletic with respect to *Heterospathius* and *Neotherospilus* (Fig. 2). The accumulation curve estimations of species richness (Fig. 3) indicates that the number of species that occur in the region has not been reached.

Twenty new species and two new genera (*Sabinita* Belokobylskij, Zaldívar-Riverón et Martínez, *Ficobolus* Martínez, Belokobylskij et Zaldívar-Riverón) have been described by AZR and collaborators from the doryctine specimens collected for this study (Table 3). An update on Zaldívar-Riverón et al. 2010) generic identification was performed. Specimens assigned to *Hansonorum* Marsh were transferred to *Notiospathius*, since the former genus is now considered its junior synonym (De Jesús-Bonilla et al. 2011). Moreover, the specimens assigned in the above study to *Barbalhoa* Marsh and *Platydoryctes* Barbalho et Penteado-Dias actually belong to *Concurtisella* and *Callihormius*, respectively.

## Discussion

The Mexican dry tropical forest is known for containing a considerably high species richness and endemicity rates for various plant and animal groups (Ceballos et al. 2010). Despite that the current available information does not allow to determine what proportion of the richness registered for insects in Mexico occurs in dry tropical forests, the gathered data for other taxa suggest that it is considerably high (Zaragoza-Caballero et al. 2010). This work represents one of various DNA barcoding species inventories that are being carried out for selected braconid subfamilies (Agathidinae, Braconinae, Rogadinae) and for other insect taxa (e.g. Coleoptera: Cerambycidae, Elateridae) in the CBS, one of which has already been published (Microgastrinae, Fernández-Flores et al. 2013).

Our updated study identified 14 additional genera and increased 53% the number of barcoding species found in the CBS with respect to the results obtained in Zaldívar-Riverón et al. 2010 preliminary study. Though most of the doryctine species that were discriminated remain undescribed, 20 of them and two new genera (*Sabinita and Ficobolus*) were already described by AZR and collaborators based on the material collected in this work. Most of these new described species belong to small, poorly known genera. The published records for the Chamela region indicate that the species richness of the Doryctinae is considerably higher than the those observed for Microgastrinae (103 spp.; Fernández-Flores et al. 2013), considered to be the second largest subfamily of Braconidae (Jones et al. 2009), and Rogadinae (27 spp.; Aguilar-Velasco 2013).

A vast species richness found for the subfamily Doryctinae was reported for Costa Rica (458 spp.; Marsh 2002, Marsh et al. 2013). This species richness, however, was reported for the whole country, whereas in our study the species richness found for the subfamily (290 spp.) was limited to about 3,000 ha. Particularly, we delimited 170 barcoding species for the genus *Heterospilus* for the CBS, whereas for Costa Rica, Marsh et al. 2013 described 280 species (Marsh et al. 2013).

Similar to our preliminary study, paraphyly of *Heterospilus* with respect to *Heterospathius* and *Neoheterospilus* was again recovered. A non-monophyletic *Heterospilus* was also recovered in a recent multi locus phylogenetic study (Wild et al. 2013). Our results therefore support that, though it is not appropriate to reconstruct phylogenetic relationships only based on a single mitochondrial marker, barcoding data represents an accessible, comprehensive system for species identification (Hebert and Gregory 2005).

## Supplementary Material

Supplementary material 1 Accumulation Curve Data Data type: Table of records
Brief description: Table containing the Process ID of specimens sampled and Barcode Index Number (BIN) used for the species accumulation curve.
File: oo_40543.xls A. Zaldívar-Riverón, C.R. Gutiérrez-Arellano, D. Gutiérrez-Arellano

XML Treatment for Allorhogas coccolobae

XML Treatment for Allorhogas crassifemur

XML Treatment for Allorhogas jaliscoensis

XML Treatment for Allorhogas marshi

XML Treatment for Allorhogas parvus

XML Treatment for Allorhogas scotti

XML Treatment for Ficobolus jaliscoi

XML Treatment for Heerz ecmahla

XML Treatment for Heerz macrophthalma

XML Treatment for Iare belokobylskiji

XML Treatment for Iare cheguevarai

XML Treatment for Iare mexicanus

XML Treatment for Lissopsius jaliscoensis

XML Treatment for Lissopsius pacificus

XML Treatment for Monarea fridae

XML Treatment for Neoheterospilus chamelae

XML Treatment for Notiospathius crypticus

XML Treatment for Notiospathius mariachi

XML Treatment for Ondigus cuixmalensis

XML Treatment for Sabinita mexicana

XML Treatment for Spathius chamelae

XML Treatment for Whitfieldiellus variegata

## Figures and Tables

**Table 1.** Sampling sites and collecting techniques employed in this study.

SITE	LATITUDE	LONGITUDE	ALTITUDE	TRAP TYPE
Near CBS	19.4985	-105.0441	92	Yellow pan trap
Camino Zarco/Chachalaca	19.4956	-105.0393	30	Sweep net
Camino Búho	19.4990	-105.0412	74	Malaise
	19.4988	-105.0404	65	Light trap
Camino Chachalaca	19.4993	-105.0383	56	Sweep net, Malise trap, Yellow pan trap
	19.4978	-105.0445	120	Sweep net
Camino Calandria	19.5084	-105.0378	120	Yellow pan trap, sweep net, light trap, Malaise trap
Eje central/Camino Calandria	19.5002	-105.0425	49	Yellow pan
	19.5039	-105.0335	52	Sweep net
Camino Calandria/Camino Chachalaca	19.5049	-105.0377	52	Sweep net
Fundación-Cuixmala/Poza del Jaguar	19.4292	-104.9796	66	Sweep net
Fundación-Cuixmala/El Sendero	19.4192	-104.9732	61	Sweep net, Malaise trap
Behind CBS dinning room	19.4978	-105.0445	120	Sweep net
Camino Ardilla	19.5042	-105.0379	39	Sweep net
Eje central	19.5085	-105.0366	32	Sweep net
Calandria(Arroyo Calandria)	19.5036	-105.0364	62	Sweep net
	19.5002	-105.0353	109	Sweep net
Camino Búho/ Chachalaca	19.4991	-105.0421	68	"O" Trap
	19.4978	-105.0444	106	Sweep net
Camino Antiguo	19.5047	-105.0367	59	Sweep net

**Table 2.** Doryctine genera and number of barcoding species identified in this study (Total 289). ^*^ Species identified only based on morphological evidence.

Genera	Barcoding species
*Allorhogas* Gahan	15
*Caenophanes* Förster	1
*Callihormius* Ashmead	14
*Coiba* Marsh	7
*Concurtisella* Roman	1
*Curtisella* Spinola	4
Doryctinus^*^ Roman	1
*Ecphylus* Förster	19
*Ficobolus* Martínez, Belokobylskij et Zaldívar-Riverón	1
*Glyptocolastes* Ashmead	8
*Heerz* Marsh	2
*Heterospathius* Barbalho et Penteado-Dias	1
*Heterospilus* Haliday	170
*Iare* Barbalho et Penteado-Dias	6
*Janzenia* Marsh	1
*Leluthia* Cameron	1
*Lissopsius* Marsh	2
*Masonius* Marsh	2
Monarea^* ^Szépligeti	1
*Neoheterospilus* Belokobylskij	3
*Nervellius* Roman	1
*Notiospathius* Matthews et Marsh	5
*Odontobracon* Cameron	2
*Ondigus* Braet, Barbalhoa et Van Achterberg	2
*Panama* Marsh	1
*Psenobolus* Reinhard	2
*Ptesimogaster* Marsh	1
*Rhaconotus* Ruthe	3
*Sabinita* Belokobylskij, Zaldívar-Riverón et Martínez	1
*Spathius* Nees	1
*Stenocorse* Marsh	3
*Vanderentiellus* Marsh	2
*Whitfieldiellus* Marsh	1
Unidentifed	4

**Table 3.** List of the new species described from specimens collected in this study

SPECIES	REFERENCE
*Allorhogas coccolobae* Martínez and Zaldívar-Riverón	Martínez and Zaldívar-Riverón 2013
*Allorhogas crassifemur* Martínez and Zaldívar-Riverón	Martínez and Zaldívar-Riverón 2013
*Allorhogas jaliscoensis* Martínez and Zaldívar-Riverón	Martínez and Zaldívar-Riverón 2013
*Allorhogas marshi* Martínez and Zaldívar-Riverón	Martínez and Zaldívar-Riverón 2013
*Allorhogas parvus* Martínez and Zaldívar-Riverón	Martínez and Zaldívar-Riverón 2013
*Allorhogas scotti* Martínez and Zaldívar-Riverón	Martínez and Zaldívar-Riverón 2013
*Ficobolus jaliscoi* Zaldívar-Riverón and Belokobylskij	Martínez and Zaldívar-Riverón 2013, Zaldívar-Riverón et al. 2014
*Heerz ecmahla* Martínez, Zaldívar-Riverón, Ceccarelli and Shaw	Zaldivar-Riveron et al. 2012
*Heerz macrophthalma* Martínez, Zaldívar-Riverón, Ceccarelli and Shaw	Zaldivar-Riveron et al. 2012
*Iare cheguevarai* Martínez, Zaldívar-Riverón and Ceccarelli	Martínez et al. 2010
*Iare mexicanus* Martínez, Zaldívar-Riverón and Ceccarelli	Martínez et al. 2010
*Lissopsius jaliscoensis* Zaldívar-Riverón, Martínez, Ceccarelli et Shaw	Zaldivar-Riveron et al. 2012
*Lissopsius pacificus* Zaldívar-Riverón, Martínez, Ceccarelli et Shaw	Zaldivar-Riveron et al. 2012
*Monarea fridae* Belokobylskij, Zaldivar-Riveron et Coronado-Blanco	Belokobyskij et al. 2014
*Neoheterospilus chamelae* Martínez et Zaldívar-Riverón	Martínez and Zaldívar-Riverón 2010
*Notiospathius crypticus* Reséndiz-Flores, Nunes and Zaldívar-Riverón	Reséndiz-Flores et al. 2014
*Notiospathius mariachi* Reséndiz-Flores, Nunes and Zaldívar-Riverón	Reséndiz-Flores et al. 2014
*Ondigus cuixmalensis* Zaldívar-Riverón, Martínez, Ceccarelli and Shaw	Zaldivar-Riveron et al. 2012
*Sabinita mexicana* Belokobylskij, Zaldívar-Riverón and Martínez	Zaldívar-Riverón et al. 2014
*Spathius chamelae* Belokobylskij and Zaldívar-Riverón	Belokobylskij and Zaldivar-Riveron 2014

## References

[B1226545] Aguilar-Velasco R. G. (2013). Diversidad de especies y asociación de parasitoidismo en las avispas de la subfamilia Rogadinae (Hymenoptera: Braconidae) de la Estación de Biología de Chamela, Jalisco. BSc Thesis. Facultad de Ciencias.

[B1191685] Belokobylskij SA (1992). On the classification and phylogeny of the Braconid wasps subfamilies Doryctinae and Exothecinae (Hymenoptera, Braconidae). Part I. On the classification, 1. Entomologicheskoe Obozrenie.

[B1355419] Belokobylskij Sergey, Zaldivar-Riveron Alejandro (2014). The genus Spathius Nees (Hymenoptera, Braconidae, Doryctinae) in Mexico: occurrence of a highly diverse Old World taxon in the Neotropics. ZooKeys.

[B792005] Belokobylskij S. A., Zaldívar-Riverón A., Quicke D. L. J. (2004). Phylogeny of the genera of the parasitic wasps subfamily Doryctinae (Hymenoptera: Braconidae) based on morphological evidence. Zoological Journal of the Linnean Society.

[B963752] Belokobyskij S A, Zaldívar-Riverón A, Coronado-Blanco J M (2014). Phylogenetic affinities of Monarea Szépligeti, 1904 (Hymenoptera: Braconidae, Doryctinae, with description of a new species from Mexico. Zootaxa.

[B1191724] Ceballos G, Martínez L, García A, Espinoza E, Bezaury-Creel J, Dirzo R (2010). Diversidad, amenazas y áreas prioritarias para la conservación de las Selvas Secas del Pacífico de México.

[B792215] Coronado-Blanco J. M., Zaldívar-Riverón A. (2014). Biodiversidad de Braconidae (Hymenoptera: Ichneumonoidea) en México. Revista Mexicana de Biodiversidad.

[B1191695] De Jesús-Bonilla Vladimir Salvador, Nunes Juliano, Penteado-Días Angélica, Csösz Sándor, Zaldívar-Riverón Alejandro (2011). A new synonym of the Neotropical parasitoid wasp genus *Notiospathius* (Braconidae, Doryctinae), with redescription of two species and description of five new species from Brazil. ZooKeys.

[B1223465] Fernández-Flores S., Fernández-Triana J. L., Martínez J. J., Zaldívar-Riverón A. (2013). DNA barcoding species inventory of Microgastrinae wasps (Hymenoptera, Braconidae) from a Mexican tropical dry forest. Molecular Ecology Resources.

[B839928] Folmer O, Black M, Hoeh W, Lutz R, Vrijenhoek RC (1994). DNA primers for amplification of mitochondrial cytochrome c oxidase subunit I from diverse metazoan invertebrates.. Molecular Marine Biology and Biotechnology.

[B792302] García Enriqueta (1988). Modificaciones al sistema de classificación climática de Köppen.

[B792148] Hawkins BA, Hochberg ME, Hawkins Bradford A., Sheehan William (1994). The implications of population dynamics theory to parasitoids diversity and biological control. Parasitoid community ecology.

[B1017307] Hebert Paul, Gregory T. Ryan (2005). The Promise of DNA Barcoding for Taxonomy. Systematic Biology.

[B863544] Hebert Paul D N, Ratnasingham Sujeevan, deWaard Jeremy R (2003). Barcoding animal life: cytochrome c oxidase subunit 1 divergences among closely related species.. Proceedings. Biological sciences / The Royal Society.

[B792191] Hebert P. D. N., Cywinska A., Ball S. L., deWaard J. R. (2003). Biological identifications through DNA barcodes. Proceedings of the Royal Society B: Biological Sciences.

[B1191675] Jones OR, Purvis Andy, Baumgart E, Quicke DLJ (2009). Using taxonomic revision data to estimate the geographic and taxonomic distribution of undescribed species richness in the Braconidae (Hymenoptera: Ichneumonoidea). Insect Conservation and Diversity.

[B863554] Kimura Motoo (1980). A simple method for estimating evolutionary rates of base substitutions through comparative studies of nucleotide sequences. Journal of Molecular Evolution.

[B792111] LaSalle J., Gauld I. D. (1993). Hymenoptera and biodiversity.

[B792339] Marshall Steven A. (1994). Terrestrial Arthropod Biodiversity: Planning a Study and Recommended Sampling Techniques: a Brief. Entomological Society of Canada Bulletin.

[B1226392] Marsh PM (2002). The Doryctinae of Costa Rica (excluding the genus Heterospilus).. Memoirs of the American Entomological Institute.

[B1023971] Marsh Paul, Wild Alexander, Whitfield James (2013). The Doryctinae (Braconidae) of Costa Rica: genera and species of the tribe Heterospilini. ZooKeys.

[B792369] Marsh P. M., Wharton R. A., Marsh P. M., Sharkey M. J. (1997). Doryctinae. Manual of the New World genera of the Family Braconidae (Hymenoptera).

[B1355429] Martínez Juan J., Zaldívar-Riverón Alejandro (2010). A new species of Neoheterospilus (Hymenoptera: Braconidae: Doryctinae) from Chamela, Jalisco, Mexico. Journal of Hymenoptera Research.

[B1355353] Martínez Juan José, Zaldívar-Riverón Alejandro (2013). Seven new species of Allorhogas (Hymenoptera: Braconidae: Doryctinae) from Mexico. Revista Mexicana de Biodiversidad.

[B1355399] Martínez J J, Ceccarelli F S, Zaldívar-Riverón A (2010). The genus Iare Barbalho and Penteado-Dias (Hymenoptera: Braconidae: Doryctinae) in Mexico, with the description of two new species. Zootaxa.

[B792228] Noguera Felipe A., Vega JH, García AN, Quesada M (2002). Historia natural de Chamela.

[B792429] Ratnasingham Sujeevan, Hebert Paul D. N. (2013). A DNA-Based Registry for All Animal Species: The Barcode Index Number (BIN) System. PLoS ONE.

[B1355409] Reséndiz-Flores Andrés, Nunes Juliano F., García-París Mario, Zaldívar-Riverón Alejandro (2014). Six new species of the parasitoid wasp genus Notiospathius (Hymenoptera: Braconidae: Doryctinae) from Mexico. Revista Mexicana de Biodiversidad.

[B792320] Rzedowski Jerzy (1978). Vegetación de México.

[B792202] Smith M. A., Rodriguez J. J., Whitfield J. B., Deans A. R., Janzen D. H., Hallwachs W., Hebert P. D. N. (2008). Extreme diversity of tropical parasitoid wasps exposed by iterative integration of natural history, DNA barcoding, morphology, and collections. Proceedings of the National Academy of Sciences.

[B792360] Wharton Robert A., Marsh Paul M., Sharkey Michael J. (1997). Manual of the New World genera of the family Braconidae (Hymenoptera).

[B1023981] Wild Alexander L., Marsh Paul M., Whitfield James B. (2013). Fast-Evolving Homoplastic Traits Are Best for Species Identification in a Group of Neotropical Wasps. PLoS ONE.

[B1191666] Yu DSK, van Achterberg C, Horstmann K (2012). Taxapad 2012, Ichneumonoidea 2011. Database on flash-drive. www.taxapad.com. Ottawa, Ontario, Canada. http://www.taxapad.com.

[B1355389] Zaldivar-Riveron Alejandro, Martínez Juan, Ceccarelli Fadia, Shaw Scott (2012). Five new species of the genera Heerz Marsh, Lissopsius Marsh and Ondigus Braet, Barbalho and van Achterberg (Braconidae, Doryctinae) from the Chamela-Cuixmala biosphere reserve in Jalisco, Mexico. ZooKeys.

[B866206] Zaldívar-Riverón Alejandro, Martínez Juan J., Belokobylkij Sergey A., Pedraza-Lara Carlos, Shaw Scott R., Hanson Paul E., Varela-Hernández Fernando (2014). Systematics and evolution of gall formation in the plant-associated genera of the wasp subfamily Doryctinae (Hymenoptera: Braconidae). Systematic Entomology.

[B792028] Zaldívar-Riverón Alejandro, Martínez Juan José, Ceccarelli Fadia Sara, De Jesús-Bonilla Vladimir Salvador, Rodríguez-Pérez Ana Cecilia, Reséndiz-Flores Andrés, Smith M. Alex (2010). DNA barcoding a highly diverse group of parasitoid wasps (Braconidae: Doryctinae) from a Mexican nature reserve. Mitochondrial DNA.

[B1191707] Zaragoza-Caballero S, Noguera FA, González-Soriano E, Ramírez-García E, Rodríguez-Palafox A, Ceballos G, Martínez L, García A, Espinosa E, Bezaury-Creel J, Dirzo R (2010). Insectos. Diversidad, amenazas y áreas prioritarias para la conservación de las selvas secas del Pacífico de México.

